# Cholesterol Metabolism Is Required for Intracellular Hedgehog Signal Transduction *In Vivo*

**DOI:** 10.1371/journal.pgen.1002224

**Published:** 2011-09-01

**Authors:** Rolf W. Stottmann, Annick Turbe-Doan, Pamela Tran, Lisa E. Kratz, Jennifer L. Moran, Richard I. Kelley, David R. Beier

**Affiliations:** 1 Division of Genetics, Department of Medicine, Brigham and Women's Hospital, Harvard Medical School, Boston, Massachusetts, United States of America; 2 Kennedy Krieger Institute, Johns Hopkins University, Baltimore, Maryland, United States of America; 3 Broad Institute of MIT and Harvard, Cambridge, Massachusetts, United States of America; Stanford University School of Medicine, United States of America

## Abstract

We describe the *rudolph* mouse, a mutant with striking defects in both central nervous system and skeletal development. *Rudolph* is an allele of the cholesterol biosynthetic enzyme, *hydroxysteroid (17-beta) dehydrogenase 7*, which is an intriguing finding given the recent implication of oxysterols in mediating intracellular *Hedgehog (Hh)* signaling. We see an abnormal sterol profile and decreased *Hh* target gene induction in the *rudolph* mutant, both *in vivo* and *in vitro*. Reduced *Hh* signaling has been proposed to contribute to the phenotypes of congenital diseases of cholesterol metabolism. Recent *in vitro* and pharmacological data also indicate a requirement for intracellular cholesterol synthesis for proper regulation of Hh activity via *Smoothened*. The data presented here are the first *in vivo* genetic evidence supporting both of these hypotheses, revealing a role for embryonic cholesterol metabolism in both CNS development and normal *Hh* signaling.

## Author Summary

The molecules and signaling pathways that regulate growth and patterning of the developing embryo are still being elucidated, and one valuable experimental approach is the use of animal models, such as the mouse. We have identified a recessive mutation in the mouse, *rudolph*, that causes abnormal forebrain development and have determined that the mutated gene encodes *hydroxysteroid (17-beta) dehydrogenase 7* gene, an enzyme necessary for cholesterol biosynthesis. Cholesterol is essential for proper signal transduction of the *hedgehog* family of proteins, key regulators of both developmental biology and tumor progression. We show that *hedgehog* signaling is diminished in our *rudolph* mutant. Our conclusions from studying this mouse mutant support two recent hypotheses in developmental biology. First, several human malformation syndromes are known to be caused by defects in cholesterol metabolism, but support linking the malformation to abnormal *hedgehog* signaling has not definitively been made. Second, while *in vitro* studies have shown that proper levels of metabolic by-products of cholesterol are necessary for proper *hedgehog* signaling, our studies offer the strongest genetic animal model evidence to support this idea.

## Introduction

*Hedgehog* (*Hh*) ligands have numerous and fundamental roles in both embryonic development [1], [2] and tumor biology [3]. Mammalian hedgehog proteins bind to the transmembrane receptor *Patched* (*Ptc*) and thereby relieve its repression of *Smoothened* (*Smo*). Intracellular transduction of *Smo* activity requires processing of GLI proteins. The primary cilium has shown to be an essential structural component for proper Hh signaling in mammals with dynamic localization of *Ptc* in response to binding of Sonic hedgehog (SHH; [4], [5], [6], [7]). Functional SHH signaling requires removal of PTC from the cilium, translocation of SMO to the cilium, and activation of SMO by an as yet unknown mechanism [5], [6], [8], [9].

It has been well established that cholesterol is an essential component of *Hh* signal transduction. Processing of the Hh ligand in the producing cell includes the covalent modification of cholesterol to the carboxyl end of the immature protein. Although cholesterol in Hh proteins is thought to facilitate the proper dispersal of SHH through the target field, it is not necessary for signal transduction [7]. More recently, however, metabolites of cholesterol and cholesterol biosynthetic pathway intermediates have been shown to have an intracellular role in SHH signal transduction. For example, pharmacological inhibition of cholesterol biosynthesis leads to defective responses to SHH ligand, independent of SHH processing, both *in vitro* and *in vivo*, with the *in vivo* effect mimicking *Shh* loss of function phenotypes [10], [11], [12]. Moreover, not only can cholesterol-derived oxysterols activate the *Hh* pathway *in vitro* [13], but treatment of cells with oxysterols has been shown to cause translocation of PTC and SMO to the cilium in the absence of SHH ligand [8].

Mutations in enzymes required for cholesterol biosynthesis are associated with a number of human diseases [14]. The best known is Smith-Lemli-Opitz Syndrome, in which patients have central nervous system (CNS) malformations, including holoprosencephaly and microcephaly, and skeletal defects (most often postaxial polydactyly) caused by mutations in *7-dehydrocholesterol reductase* (DHCR7) [15]. Other disorders of cholesterol biosynthesis include desmosterolosis, lathosterolosis, X-linked dominant chondrodysplasia punctata (CDPX2), CHILD syndrome (congenital hemidysplasia with icthyosiform erythroderma or nevus and limb defects) and Greenberg skeletal dysplasia. Overlapping features of these disorders include abnormalities in the CNS, facial dysmorphisms, and skeletal defects, often including polydactyly or other digit patterning defects. Mouse models also exist for many of these disorders and have similar defects. The frequent occurrence in these human syndromes and mouse mutants of defects in neurodevelopment, craniofacial morphogenesis and skeletal growth and patterning has led to the proposal that abnormal *Hh* signaling may be the root cause of these embryological defects. This speculation is based principally on genetic experiments that have shown a role for *Hh* signaling in the development of all of these tissues, and the known role of sterol metabolism in *Hh* signal transduction. Despite this, there is relatively little direct evidence from these mouse models for a defect in *Hh* signaling.

Here we describe the phenotype of the *rudolph* mouse mutant, an ethyl-nitrosourea (ENU)-induced mutation in *hydroxysteroid (17-beta) dehydrogenase 7* (*Hsd17b7*), which was the last enzyme of the cholesterol biosynthetic pathway to be identified and one of four proteins of the sterol-4-demethylase complex [16]. *Rudolph* mutants have severe developmental abnormalities in several tissues including the brain and appendicular skeleton. We find that tissues from *rudolph* mutants have an abnormal sterol profile consistent with impaired activity of the sterol-4-demethylase complex. We further demonstrate that the *rudolph* mutant has deficient responses to *Hh* signaling, both *in vivo* and *in vitro*. These results support the recently proposed model that functional intracellular sterol metabolism is required for proper cilia-mediated activation of the *Hh* signaling pathway.

## Results

### *Rudolph* mutants show defective growth and patterning of the CNS and appendicular skeleton

**Figure 1:**
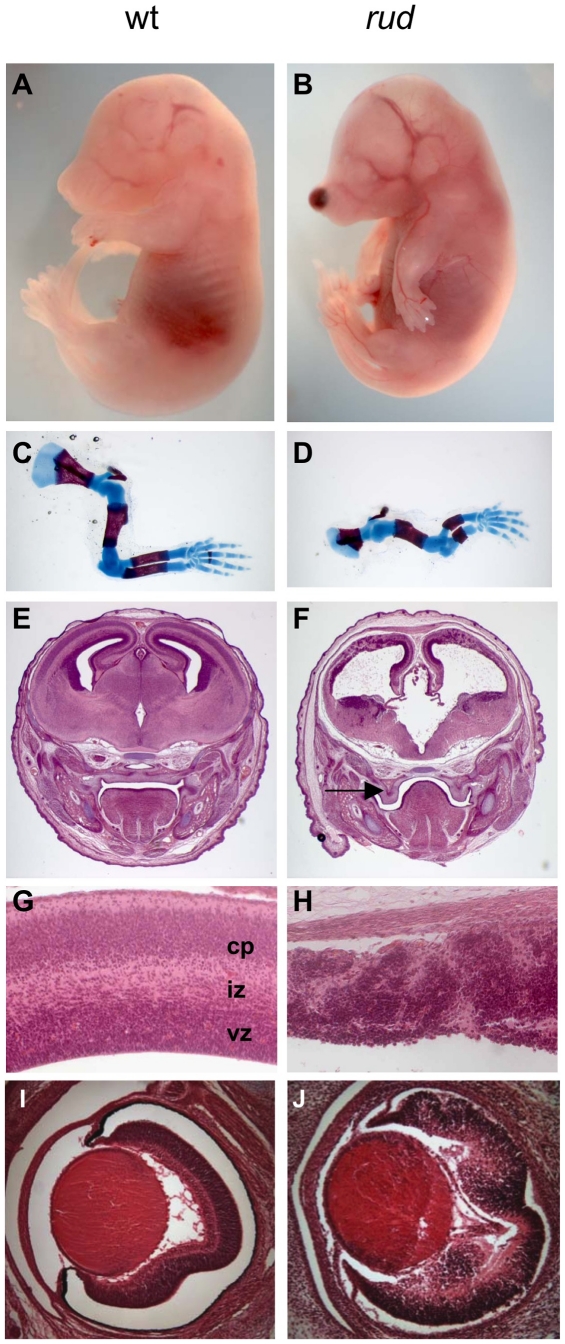
Phenotypic characterization of *rudolph* mutants. *Rudolph* mutants (B) are readily distinguishable from wild-type (A) littermates at E16.5 by their shortened, curved forelimbs. Some mutants have accumulations of blood in nasal blebs (B). Skeletal preparations of E16.5 limbs show significantly shorter long bones in mutants (D). The mutant forebrain at E16.5 (F) is severely affected with a reduction in tissue throughout. Cleft palate is also noted in mutants (arrow in F). While the wild-type cortex (G) has a readily identifiable ventricular zone (vz), intermediate zone (iz), and cortical plate (cp), closer examination of the dorsal cortex in the mutant reveals significant disorganization (H). Similar disorganization is seen in the *rud* retina (J).

We recently recovered the *rudolph* mutation via an ENU mutagenesis screen designed to identify recessive mutations affecting development of the mammalian forebrain. *Rudolph* mutants were first ascertained by a blood spot on the end of the nose and their abnormally curved forelimbs (Figure 1B). The precursor to this nasal phenotype was sometimes evident at earlier stages as a blebbing of the craniofacial epithelium (Figure S1A). Examination of the embryonic skeleton revealed that all long bones of the appendicular skeleton were significantly shorter than those of wild-type littermates while the axial skeleton and ribs appeared normal. (Figure 1D, Figure S2, Table S1). Further analysis of the embryos revealed severe defects in CNS development. The telencephalic tissue was markedly reduced in size and highly disorganized in mutants at embryonic day (E) 16.5 (Figure 1F, 1H). Mutants had a smaller neurogenic ventricular zone and clumps of cells in the developing cortical plate. Similar defects were seen in the E16.5 retina and spinal cord (Figure 1J, Figure S3B). Initial cortical morphogenesis appeared largely normal (Figure S3D, S3F).

**Figure 2:**
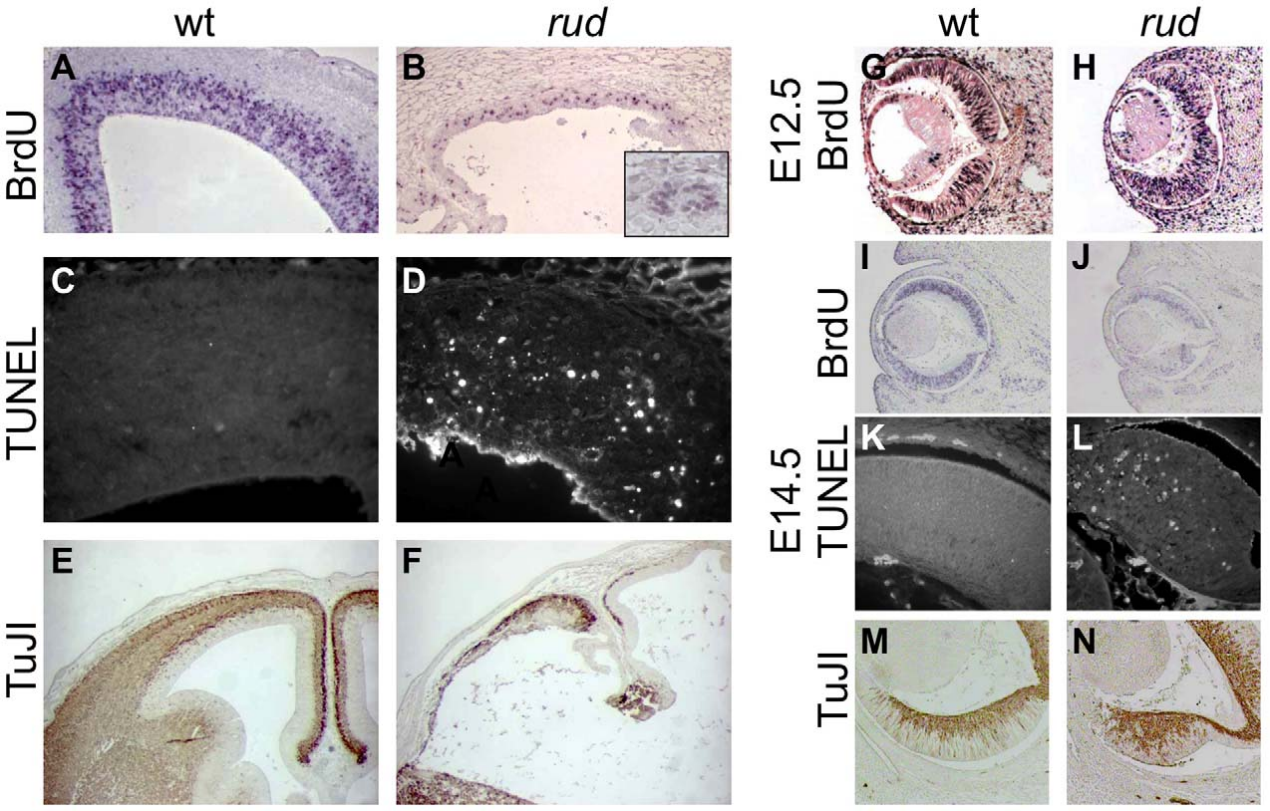
Telencephalic and retinal development are abnormal in *rudolph* mutants. (A,B) BrdU labeling and immunohistochemistry shows a significant decrease in dividing cells in the *rudolph* cortex (A) as compared to the wild-type (A). The inset in B highlights the groups of dividing cells sometimes seen away from the ventricular zone. (C,D) Analysis of cell death using the TUNEL assay reveals a large increase in cell death in mutant tissue (B) with an enrichment of the death at the ventricular surface. (E,F) Neuronal differentiation as measured by TuJI immunohistochemistry is dramatically decreased in rud mutants (F). (G,H) BrdU labeling at E12.5 does not indicate a significant difference in rates of cell division in the *rudolph* retina at E12.5, but a significant decrease is seen at E14.5 (J). Similar to cortical development, the decreased proliferation is accompanied by an increase in apoptosis as shown by the TUNEL assay (K,L). Neuronal differentiation in the mutant retina is disorganized in mutant tissue (M) as compared to wild-type (N).

To further characterize the *rudolph* phenotype, we performed a molecular analysis of the cortical phenotype. We assessed cell proliferation at E14.5 by BrdU treatment of pregnant dams or immunostaining of embryos with Ki67 and found a marked decrease in proliferation in mutants (Figure 2B). Some of the mitoses we detected were seen as foci of BrdU-positive cells (inset in Figure 2B). We thus interpreted the clusters of cells seen histologically in the *rud* cortex to be neurogenic foci. To determine the cause of the reduced neuronal tissue, we assayed apoptosis at E14.5 using the TUNEL reaction and found increased levels of cell death in the mutant tissue, distributed throughout the cortex and enriched along the ventricular surface (Figure 2D). An increased level of cell death was not seen in non-neural tissue (data not shown). Decreased, disorganized neuronal proliferation and increased cell death were also evident at E12.5 (data not shown). Immunohistochemistry for TuJI to identify differentiated neurons at E14.5 showed a marked decrease in differentiation in mutants compared to wild-type (Figure 2F). In addition, foci of TuJI immunoreactivity appeared in regions of no differentiation, consistent with the disorganization of the cortex seen histologically. Disorganized proliferation was also evident in the developing *rudolph* retina, where we observed similarly abnormal neuronal differentiation, decreased cell proliferation and increased apoptosis, but at different stages of development. Whereas at E12.5 we saw no significant decrease in proliferation or apoptosis between wild-type and mutant (Figure 2H; data not shown), at E14.5 we found a decrease in BrdU incorporation in the mutant retina (Figure 2J) and an increase in apoptosis (Figure 2L). Furthermore, the pattern of neuronal disorganization we saw in the *rud* cortex was similarly evident in the *rud* retina at E14.5 (Figure 2N).

### Genetic background affects the *rudolph* phenotype

The phenotypes of the *rudolph* mutants exhibit a variability in severity that appears to be dependent on genetic background. For example, we noted increased blebbing on the developing head and limbs in embryos that came from a mixed background (Figure S1B–S1D). Embryos from our ENU screen have a mixed genetic background with contributions coming from both A/J (the mutagenized strain) and FVB mice (introduced as part of the outcross for mapping purposes). In this A/J; FVB background, embryos were recovered in approximately Mendelian ratios from E10.5–E18.5 (Table S2). No mutants have been recovered after birth, suggesting they are among the stillborn fetuses. Upon introducing the B6 background, the number of mutant embryos did not decrease significantly, but we then began to see a number of dead embryos from E11.5 and older (7.8%), an increase in the severity of the nasal blebbing (Figure S1A–S1C: 14.8%) and limb patterning defects (10.4%). Further introgression into the B6 background resulted in no significant decrease in recovery of mutant embryos but a large increase in the incidence of more severe blebbing (48.5% in pooled N1, N2, and N3B6 mice). Backcrossing to FVB rescued this defect, and blebbing is essentially absent in N3 FVB mice. The reduced fecundity of 129 mice limited our analysis of this genetic background, preventing any definitive comments on modifiers on the 129 background (Table S2).

### *Hsd17b7* is the gene mutated in *rudolph* embryos

**Figure 3:**
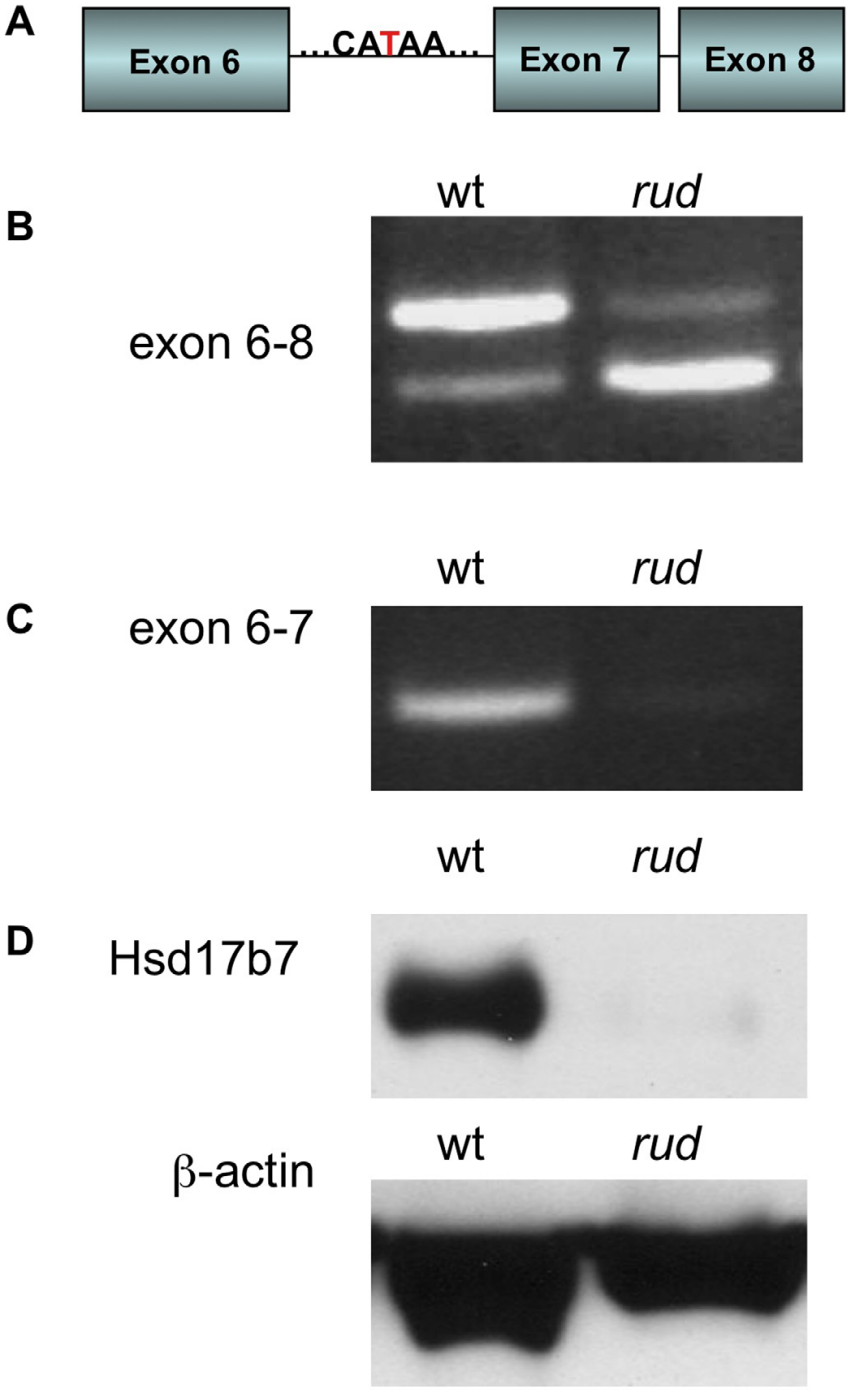
*Hsd17b7* is the gene carrying the *rudolph* mutation. (A) Sequencing of the *Hsd17b7* gene revealed a thymidine to adenosine mutation in the intron of *rudolph* mutants between the sixth and seventh exons. (B,C) RT-PCR analysis of wild-type and mutant transcripts with primers for exons 6–8 of the *rudolph* transcript give two PCR products. The larger species is found in low quantities in the *rud* mutant tissue. A smaller species, which lacks the seventh exon, was also amplified. RT-PCR with primers for the sixth and seventh exon results in very little amplification of mutant cDNA. (D) Immunoblotting from embryo lysates shows very little protein in *rudolph* mutants as compared to wild-type.

We initially mapped the *rudolph* mutation to Chromosome 1 using a whole-genome 768-marker single nucleotide polymorphism (SNP) panel [17]. Examination of the 255 predicted and known genes in the region suggested *Hsd17b7* as a candidate for further analysis because of its known function in cholesterol metabolism and its reported expression pattern in limb buds and the developing nervous system [16]. Sequencing of *Hsd17b7* revealed a point mutation in the sixth intron, 27 base pairs upstream of the intron-exon boundary (Figure 3A). We analyzed transcripts by RT-PCR with primers spanning exon 7 and found the predominant PCR product in mutant tissue to be smaller than that of wild type (Figure 3B). Sequencing of this product revealed a precise excision of the seventh exon in the smaller PCR species. The loss of exon 7 was confirmed with primers in the sixth and seventh exons (Figure 3C). Interestingly, a small amount of this truncated transcript was present in wild-type cDNA, and, conversely, mutant tissues retained a very small fraction of the wild-type transcript (Figure 3B, 3C). We hypothesize that these RT-PCR products represent two naturally occurring forms of the *Hsd17b7* transcript and that the *rudolph* ENU mutation affects the ratio of their abundances. The phenotypes we observe in the *rudolph* mutants appear to be somewhat tissue specific and have differing expressivity in different strains. However, the variation in the cDNA splicing pattern does not differ among different tissues examined (heart/lung, limbs, brain, or whole embryo) or depend on varying genetic backgrounds, suggesting that tissue specific transcription and variation in genetic background account for the variability in phenotype (Figure S1E).

Loss of the seventh exon of *HSD17B7* is predicted to encode a protein with an in-frame deletion of 19 amino acids. To assess the effect of this splicing mutation, we tested HSD17B7 expression by Western immunoblot analysis and found only trace levels of protein in mutant tissue lysates (Figure 3D). Although the deleted seventh exon is part of a putative endoplasmic reticulum anchoring sequence, *in vitro* expression of a *rud-GFP* fusion protein results in deficient protein rather than mislocalization, suggesting that the *rudolph* deletion generates an unstable protein product (Figure S4). Embryos homozygous for a null allele of *Hsd17b7* generated from a 129 genetic background do not survive past E10.5, suggesting the *rudolph* allele is likely a hypomorph [18], [19].

### Sterol profiles are abnormal in *rudolph* tissues

**Figure 4:**
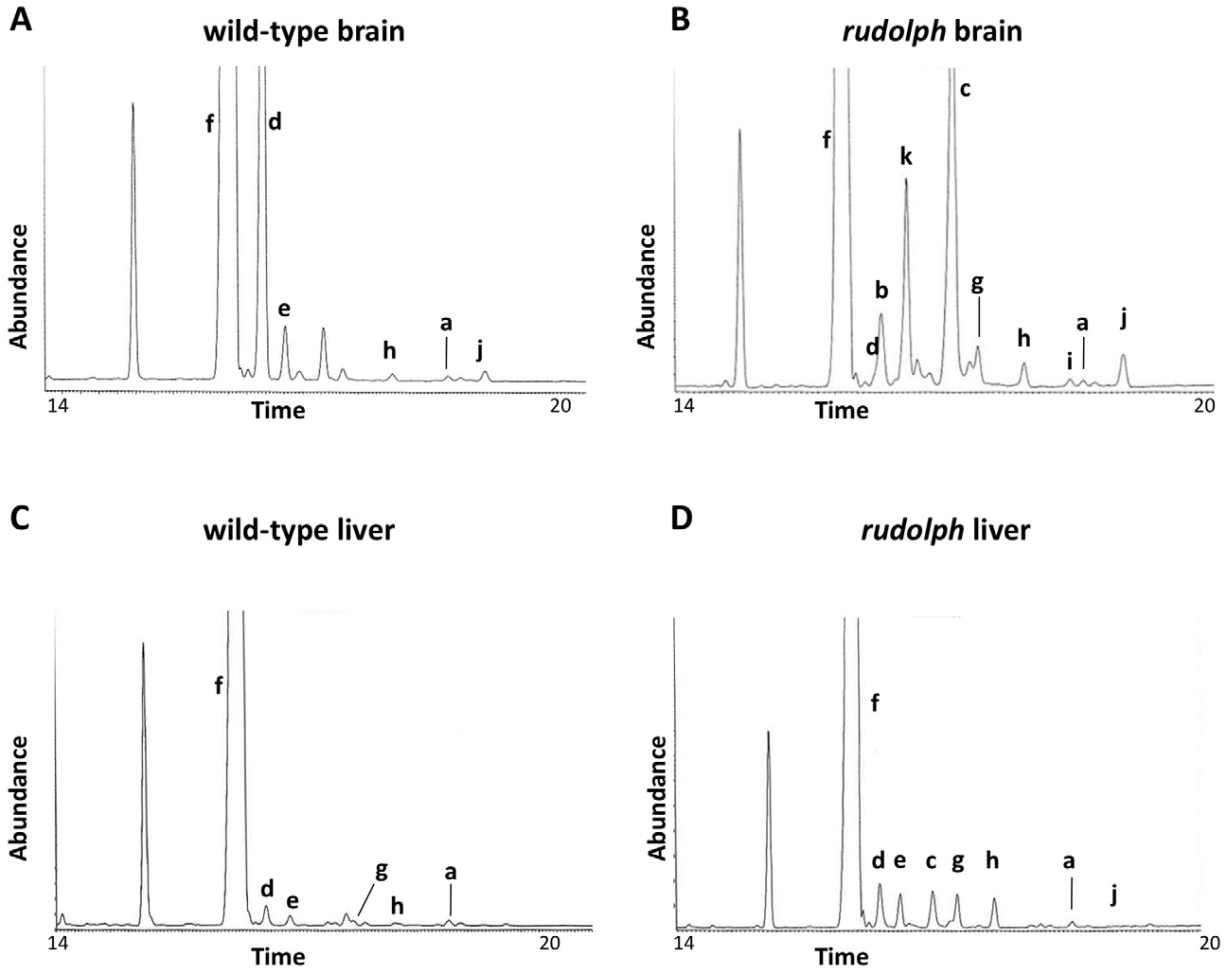
Sterol profiles. Brain (A,B) and liver (C,D) tissue from wild-type (A,C) and *rudolph* (B,D) E14.5 embryos were used to create sterol profiles by gas chromatography-mass spectrometry. Some compounds identified include lanosterol (a), zymosterone (b), 4α-methyl-zymosterone (c), desmosterol (d), lathosterol (e), cholesterol (f), 4α-methyl-5α-cholest-8-en-3β-ol (g), 4α-methyl-5α-cholest-7-en-3β-ol and 4α-methyl-cholesta-8(9),24-dien-3β-ol (h), 4,4′-dimethyl-5α-cholest-8-en-3β-ol (i), 4,4-dimethyl-5α-cholest-8(9),24-dien-3β-ol (j), and methylcholest-7-en-3-one (tentative) (k).

We analyzed the sterols present in liver and brain tissue from wild-type, heterozygous, and *rudolph* embryos at E12.5 by gas chromatography-mass spectrophotometry (Figure 4) and found marked differences between wild-type and mutant tissues. The identified abnormalities in methylsterol abundances are consistent with reduced function of the *Hsd1b7* enzyme in *rudolph* brain tissues (Table 1). Sterol species upstream of Hsd17b7 activity were present in increased amounts, the most prominent of which were the HSD17B7 substrates zymosterone and 4methyl-zymosterone, and a third ketosterol tentatively identified as methylcholest-7-en-3-one. Various mono- and dimethylsterols that do not normally accumulate in wild-type tissues were also present in increased amounts, including 4α-methyl-5 α-cholest-8-en-3β-ol, 4 α-methyl-5 α-cholest-7-en-3 β-ol, 4 α-methyl-cholesta-8(9),24-dien-3 β-ol, and 4,4-dimethyl-5 α-cholest-8(9),24-dien-3 β-ol. Desmosterol, a compound downstream of Hsd17b7, was reduced. The predominance of zymosterone (5 α-cholesta-8,24-dien-3-one) and 4 α-methylzymosterone in the brain compared to liver is in keeping with the known decreased activity of desmosterol reductase in the brain, especially fetal brain, and the retention of the 24-unsturated bond in certain sterol species in the normal brain. In wild-type brain, desmosterol (5 α-cholesta-5,24-dien-3β-ol) is the most abundant 24-unsaturated sterol.

**Table 1:** Analysis of sterols present in brain tissue.

	E14.5 Brain	
	wt	*rud*
**Upstream of Hsd17b7**		
Lanosterol (a)	0.19	0.30
4α-methyl-5α-cholest-8-en-3β-ol (g)	0.13	5.86
4α-methyl-5α-cholest-7-en-3β-ol (h)	0.04	0.58
4α-methyl-cholesta-8(9),24-dien-3β-ol	0.31	1.95
4,4′-dimethyl-5α-cholest-8-en-3β-ol (i)	0.03	0.44
4,4′-dimethyl-5α-cholest-8(9),24-dien-3β-ol (j)	0.49	2.16
methylcholest-7-en-3-one (k)*	0.000	7.164
**Substrates of Hsd17b7**		
Zymosterone (b)	0.000	2.185
4α-methylzymosterone (c)	0.021	14.787
**Downstream of Hsd17b7**		
Desmosterol (d)	15.65	1.07
Cholesterol (f)	80.48	59.88

Sterols levels are quantified as percent of total sterols in tissue. Values from heterozygous embryos were very similar to those found in wild-type.

*Tentative identification.

### Hedgehog signaling is disrupted in the *rudolph* mutant central nervous system

**Figure 5:**
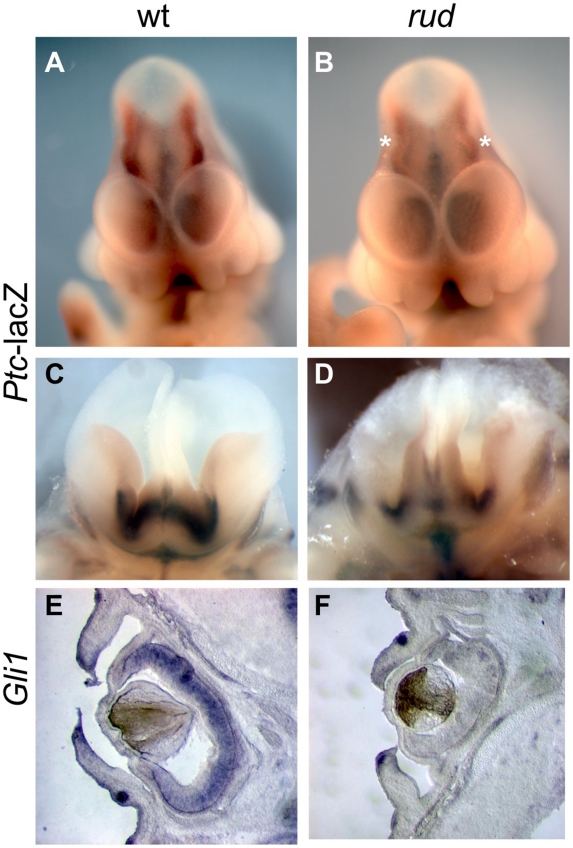
*Sonic hedgehog* signal transduction is abnormal in *rudolph* embryonic nervous system and cells. (A–D) *Rudolph-Patched lacZ* embryos at E11.5 (A,B) and E14.5 (C,D) show less *Ptc-lacZ* activity in *rudolph* mutant brain tissue (B,D) as compared to wild-type (A,C). Asterisks in B indicate the midbrain in the mutant where Ptc-lacZ is particulary reduced compared to wild-type. Section *in situ* hybridization shows less *Gli1* expression in the *rudolph* retina at E14.5 (F) than in wild-type (E).

In view of the important role of *Hh* signaling in patterning of the limbs, face, and brain, and the genetic and cellular evidence for a role of cholesterol metabolism in this pathway, we hypothesized that *Hh* signaling is perturbed in the *rudolph* mutant. Furthermore, recent evidence specifically implicates intracellular sterols in the regulation of the subcellular localization of the *Hh* signaling components, Patched and Smoothened, supporting the possibility that an abnormal sterol profile in *rudolph* mutants could disrupt *Hh* signaling [5], [6], [8]. To assess this, we generated mice homozygous for the *rudolph* mutation that carried the *Patched-lacZ* gene, a transcriptional target of *Gli2* and thus a reporter of Shh signaling activity. In these embryos, we found reduced levels of *Ptc-lacZ* in the developing brain at both E11.5 and E14.5 (Figure 5B, 5D; Figure S5; data not shown). We also noted decreased expression of another *Shh* target gene, *Gli1*, in the retina and brain of *rudolph* mutants at E14.5 (Figure 5F, Figure S5). Furthermore, analysis using quantitative RT-PCR demonstrated reduced *Ptc* mRNA in *rudolph* brain tissue compared to wild-type (63% of wild-type, p = 0.053, data not shown). All tissues with reduced SHH target gene expression have severe morphological abnormalities in the *rudolph* mutant and are known sites of *Hh* signaling. Because dorsal-ventral patterning of the neural tube also requires SHH signaling, we examined the dorsal-ventral character of the *rudolph* neural tube and found that, at both E10.5 (Figure S6A–S6N) and E12.5 (Figure S6O–S6BB), all immunohistochemical markers for cell fate we tested showed normal patterns of expression along the dorsal-ventral axis of the *rudolph* neural tube.

### Hedgehog signaling is disrupted in the *rudolph* mutant skeleton

**Figure 6:**
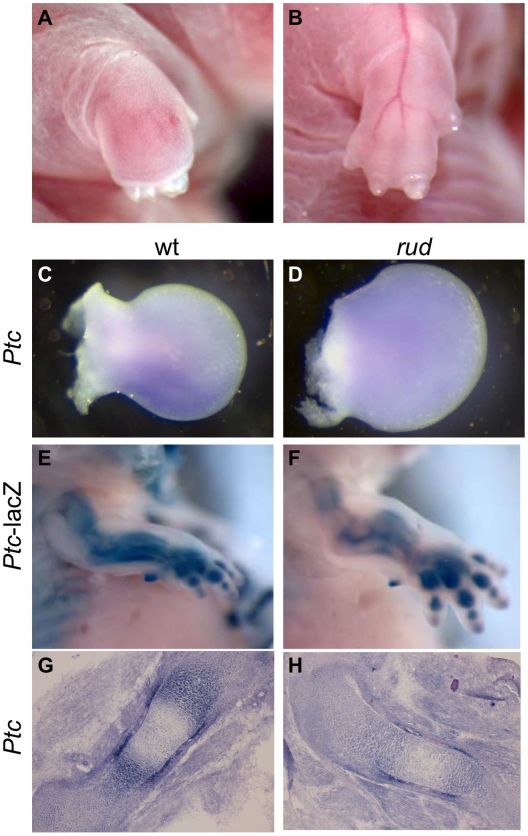
*Sonic hedgehog* signal transduction is abnormal in *rudolph* skeletal elements. (A,B) Embryos from a mixed background show an incompletely penetrant limb patterning defect. Whole mount *in situ* hybridization shows a *Ptc* domain in the posterior portion of the developing wild-type limb bud (C), which can be lost in *rudolph* mutants (D). *Hedgehog* signaling is also decreased in the developing long bones of *rudolph* mutants (F,H) compared to wild-type (E,G) as shown by *Ptc-lacZ* (E,F) or section *in situ* hybridization (G,H).

We also observed limb patterning defects in mice from a mixed A/J, FVB, B6 (Figure 6A, 6B, Table S2). As *Hh* signaling is important for proper patterning, we examined *Hh* signaling in the developing limb bud. Normal patterning of the limb results in elevated *Shh* signaling in the posterior portion of the developing limb bud as compared to the anterior. We observed *Ptc* expression in *rud* mutants (n = 3) from a mixed background using both *Ptc-lacZ* expression and whole mount *in situ* hybridization and found one embryo with reduced *Ptc* expression in the posterior limb bud (Figure 6D) as compared to littermate control (Figure 6C). The variable penetrance of the limb patterning phenotype is consistent with an incompletely penetrant reduction in *Shh* activity in the developing limb bud. *Hh* signaling is also involved in long bone growth (where the relevant ligand is *Indian hedgehog*). We also see reduced expression of *Ptc* in the developing limb of *rud* mutants using either the *Ptc-lacZ* allele (Figure 6F) or an in situ riboprobe for *Ptc* (Figure 6H).

### Hedgehog signaling is reduced in vitro upon reduction of *Hsd17b7*

**Figure 7:**
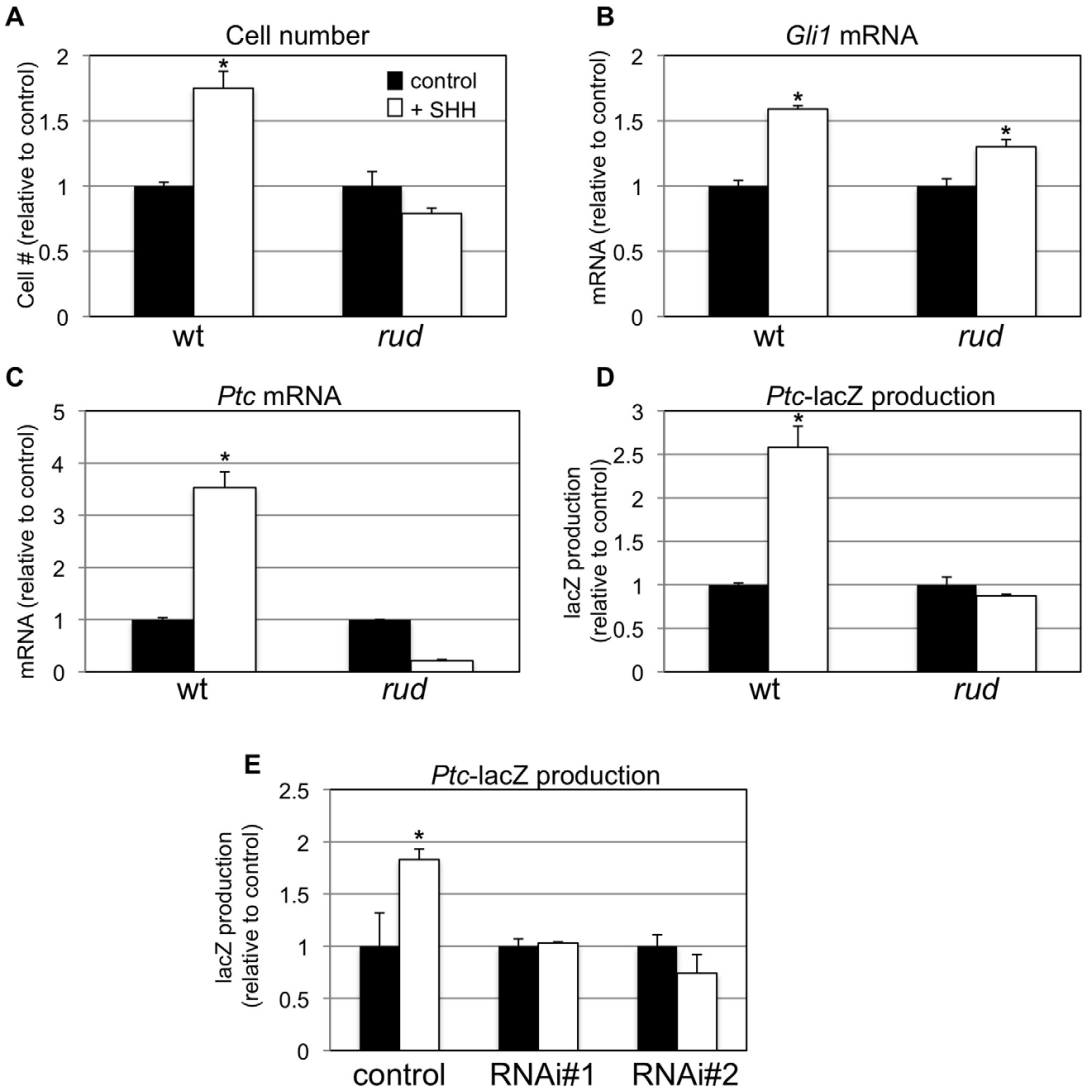
*Sonic hedgehog* signal transduction is abnormal in *rudolph* cells. *In vitro* analysis also shows a decreased response to hedgehog protein in mouse embryonic fibroblasts (MEFs). Wild-type, but not mutant, MEFs show increased cell growth upon treatment with SHH (A; *p = 0.001 for wt; n = 4). Quantitative RT-PCR analysis also shows a reduced response of *Gli1* (B; *p = 0.002 for wt, p = 0.01 for *rud*; n = 3) and *Patched* (C; *p = 0.001 for wt; n = 3) upon SHH treatment in mutant MEFs. β-galactosidase accumulates in SHH-treated wild-type;*Ptc-lacZ* MEFs, but not *rud;Ptc-lacZ* MEFs (D; *p = 0.005 for wt; n = 4). (E) PzP53Med (*Ptc-lacZ;p53* medulloblastoma) cells infected and clonally selected with control plKO.1 lentivirus respond to SHH treatment with accumulation of β-galactosidase (* p = 0.07 for wt; n = 4). Two independent clonal lines infected with *Hsd17b7* RNAi constructs do not respond as robustly to SHH treatment.

To further determine if *Hsd17b7* expression affects SHH signaling, we generated primary mouse embryonic fibroblasts (MEFs) from wild-type and *rudolph* embryos and assessed their response to added SHH protein. In wild-type MEFs, treatment with SHH protein resulted in increased cell proliferation and increased *Ptc* and *Gli* mRNA levels, which is consistent with the known role of SHH as a mitogen in several systems and with *Ptc* and *Gli* being direct targets of SHH signaling. In contrast, the effects of SHH treatment were blunted in mutant cells (Figure 7A–7C). We also generated MEFs from wild-type;*Ptc*-lacZ and *rudolph*;*Ptc*-lacZ embryos to measure SHH transcriptional activity via the accumulation of β-galactosidase. In this assay, wild-type;*Ptc*-lacZ MEFs responded to SHH treatment with increased β-galactosidase production, whereas *rudolph*;*Ptc*-lacZ MEFs did not (Figure 7D). Together these data suggest that *rudolph* mutant mice have reduced intracellular signal transduction distal to the binding of SHH ligand in the SHH signaling cascade.

In a parallel approach, we used Pzp53MED cells [20], which are SHH-responsive cells carrying the *Ptc*-lacZ allele, to assess the role of *Hsd17b7* in SHH signal transduction. We used lentiviral infection followed by clonal selection with plasmids for RNAi knockdown of *Hsd17b7* to create cell lines with reduced levels of *Hsd17b7* (approx. 80% reduced, data not shown) and then treated the lines with recombinant SHH, as done with the MEFs. Two independent RNAi clones did not induce significant β-galactosidase production upon SHH treatment while the control cell line responded robustly (Figure 7E).

### Smoothened localization to the primary cilium is unaffected in the *rudolph* mutants

**Figure 8:**
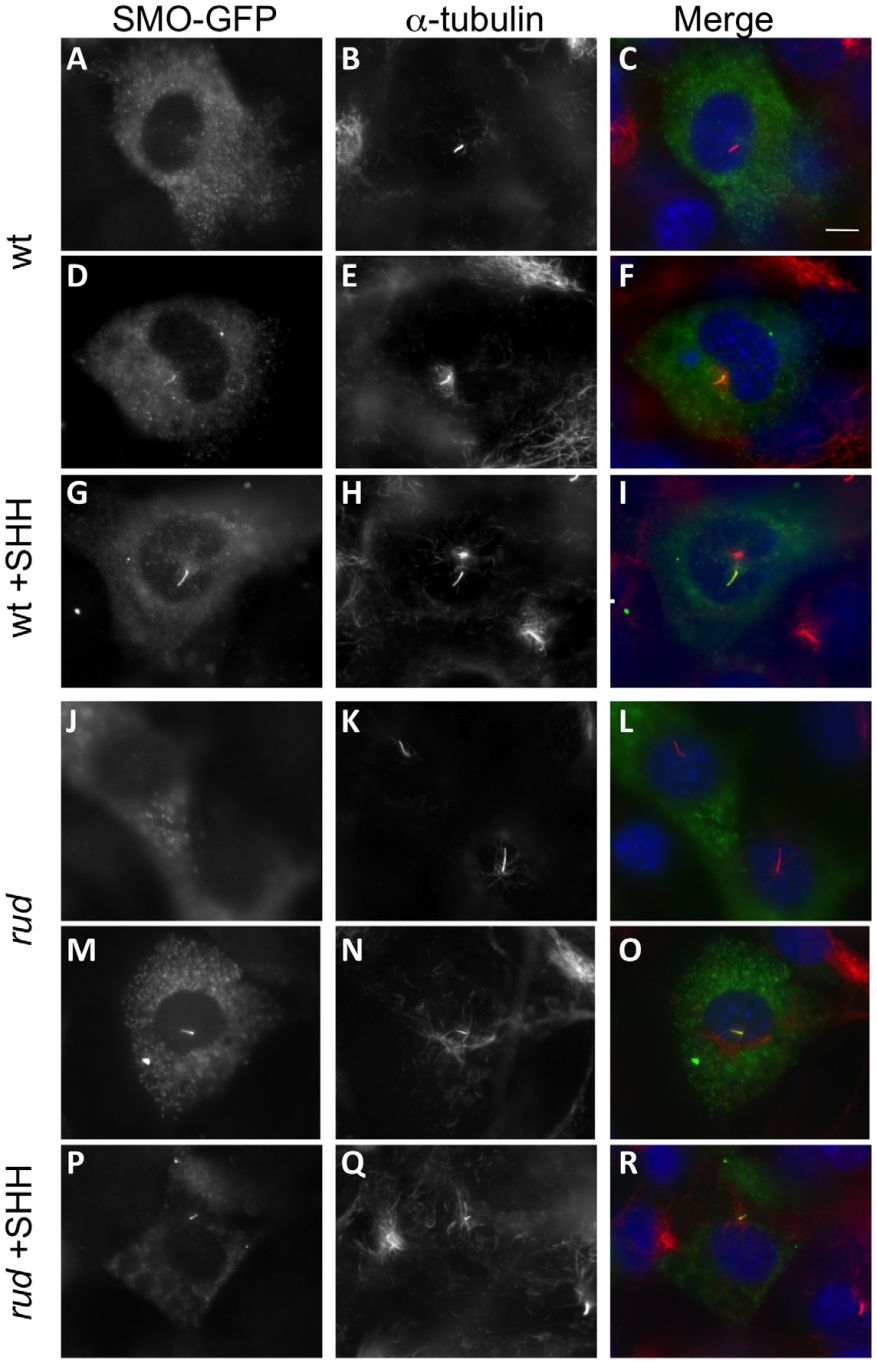
SMO-GFP localization to the primary cilium is not affected in *rudolph* mutant MEFs. Wild-type (A–I) and *rudolph* (J–R) mutant MEFs were transfected with a SMO-GFP plasmid and acetylated α-tubulin immunocytochemistry was used to identify the primary cilium. SMO-GFP in untreated cells is seen outside the cilium (A–C, J–L), accumulating at the base of the axoneme (not shown), or throughout the cilium (D–F, M–O). Treatment with SHH protein localizes SMO-GFP throughout the cilium in the majority of cells observed (G–I, P–R). All images were taken at the same magnification and the scale bar in C represent 10 µm.

The decreased response to SHH protein *in vitro* shows that the signaling defect is downstream of ligand binding to cell surface receptors. Recent data have shown that treatment with oxysterols can cause a change in the subcellular localization of PTC and SMO protein [8], which is necessary but not sufficient for activation of the SMO protein [6], [21]. The *Hsd17b7* phenotype may be caused by dysregulated sterol biosynthesis affecting the localization and/or active state of the *Smo* receptor. We therefore examined the localization of a SMO-GFP fusion protein in wild-type and *rudolph* MEFs upon treatment with SHH, but found no decreased mobility of the SMO-GFP to the cilia in the mutant MEFs (Figure 8). Wild-type MEFs without SHH treatment had SMO-GFP throughout the primary cilia in only a subset of cells examined (43.3%: n = 29/67 ciliated, transfected cells in two independent experiments). Upon addition of SHH, SMO-GFP was found throughout the cilium in the majority of cells examined (89.6%; 65/73 cells). Mutant MEFs behaved similarly, with untreated cells having 38.7% SMO-GFP positive cilia (24/62) and SHH treatment leading to 86.7% (72/83) of cells with SMO-GFP throughout the cilium. In addition to the increase in cells with SMO-GFP throughout the cilium upon SHH treatment, we also note that untreated cells often had SMO-GFP largely at the base of cilium. SHH treatment resulted in very few cells showing SMO-GFP localization to the base of the cilium, but rather throughout the length of the cilium. Taken together, these data suggest that the *rudolph* mutation does not affect the localization of SMO within the primary cilium in response to SHH treatment.

## Discussion

In this report, we describe the *rudolph* mouse mutant phenotype, which is caused by a mutation in the cholesterol biosynthetic enzyme *Hsd17b7*, the 3-ketosteroidreductase element of the sterol-4-demethylase complex. The *rudolph* phenotype includes marked abnormalities in the development of the nervous system and appendicular skeleton, which correlate with a decreased effectiveness of SHH signaling both *in vivo* and *in vitro*. Because sterols have been shown to affect SMO subcellular localization, we tested the movement of SMO to the primary cilium in response to SHH protein and found no defect in SMO trafficking. This suggests that the abnormality in *rudolph* affects the activation of SMO, resulting in a decreased response to SHH ligand binding.

### Sterols in *rudolph* mutants

Our study of the sterols present in brain tissues from the mutant mice is consistent with reduced *Hsd17b7* enzymatic function, as we observe an increase in compounds of the cholesterol biosynthetic pathway upstream of the *Hsd17b7* enzyme. While no patient has yet been identified with a defect in *Hsd17b7*, increased levels of mono and dimethyl sterols have been reported in plasma and/or skin of patients with mutations in two other genes of the sterol-4-demethylase complex, *SC4MOL* (sterol C4-methyloxidase like) and *NSDHL* (NAD(P)H steroid dehydrogenase-like protein) [22], [23].

To explain the phenotype of the *rudolph* mouse we considered several possible metabolic effects, including 1) a decrease in the cellular level of cholesterol, 2) a decreased level of another product of *Hsd17b7* enzymatic function, and 3) teratogenic effects of high levels of the cholesterol precursors detected in our study. Several lines of evidence suggest the last to be the major cause of the phenotype we observe. If simply a deficiency of the end-product, cholesterol, caused the *rudolph* phenotype, one might expect mice carrying mutations in the cholesterol biosynthetic pathway to resemble each other. This is not the case, since, despite some phenotypic overlap in adjacent disorders in the pathway, overall there are significant phenotypic differences across the spectrum of mouse models of human cholesterol biosynthetic disorders [24]. The best-characterized disorder of cholesterol biosynthesis is Smith-Lemli-Opitz syndrome [25], [26], caused by mutations in *DHCR7*, which encodes the 7-dehydrocholesterol reductase that converts 7-dehydrocholesterol to cholesterol [15], [27], [28]. Two mouse models with null alleles of *Dhcr7* have abnormal phenotypes including cleft palate, but lack the striking brain phenotypes we found in the *rudolph* mutant [29], [30]. The *Dhcr7* mutant mice have decreased cholesterol and increased 7-dehydrocholesterol levels in serum and tissues [29]. The enzyme immediately preceding *DHCR7* is *SC5DL* (sterol C5-desaturase-like) which is deficient in human patients with lathosterolosis [31]. A null *Scd5* allele in the mouse is a neonatal lethal with craniofacial and limb defects and decreased cholesterol similar to those of the *Dhcr7*-deficient mouse, but with increased levels of lathosterol (the substrate of *Sc5d*) in all tissues [32]. Given that the *Dhcr7* and *Scd5* mouse models have a significant decrease in cholesterol levels, which is not apparent in the *rudolph* mutant, and that their phenotypes do not resemble the *rudolph* phenotype, we conclude that cholesterol deficiency does not cause the distinctive embryological abnormalities of the *rudolph* mouse.

*Nsdhl*, another element of the sterol-4-demethylase complex, is the enzyme immediately preceding *Hsd17b7* in the canonical cholesterol biosynthetic pathway [33]. *NSDHL* mutations in humans cause CHILD syndrome (congenital hemidysplasia with ichthyosiform erythroderma and limb defects), a rare X-linked dominant disorder with presumed lethality for CHILD causing alleles [34] and, with hypomorphic NSDHL mutations, CK syndrome, a form of X-linked mental retardation [22]. Mutations in *Nsdhl* are found in the *Bare patches* (*Bpa*) and *Striated* (*Str*) mice [35]. Analysis of tissue samples from *Bpa/Str* females showed an accumulation of 4-methyl and 4,4′-dimethyl sterol intermediates. Human mutations in EBP cause X-linked dominant chondrodysplasia punctata (CDPX2, Conradi-Hunermann syndrome [36]). Patients with CDPX2 usually have normal plasma total cholesterol levels but increased levels of other sterols, including 8-dehydrocholesterol and cholesta-8(9)-en-3β-ol [36], [37], [38], [39]. *Tattered* (*Td*) carries a missense mutation in the *Ebp* gene and phenotypically resembles the *rudolph* mutation most among all the known cholesterol biosynthetis mouse mutants. Male *Td* embryos die between E12.5 and birth and have defects in the skeleton and brain similar to those we describe here [40]. The sterol profile of heterozygous female mice includes elevated 8-dehydrocholestrol and choles-8(9)-en-3β-ol. All of these findings combined with our data suggest a model in which the accumulation of specific sterols leads to the defects we observe. One of the effects these inhibitory sterols may be to dampen the intracellular response to *Shh* signaling.

### *Rudolph* mutants show similarities to other loss-of-function Shh phenotypes

Although *rudolph* mutants lack some of the classic features of the *Shh* null mice, such as holoprosencephaly, other more specific ablations of SHH signaling have some features resembling the *rudolph* phenotype. In particular, the skeletal defects we observe in the *rudolph* mutant are similar to those described in the *Indian hedgehog* (*Ihh*) and *dispatched-1* (*Disp1*) loss of function mice and the conditional ablation of *Smo* from the developing skeleton [41], [42], [43]. As *Ihh* is the most active *Hh* ligand in development of long bones, we suggest that the similarities between *rud* and the HH-signaling mutants reflect the conservation of intracellular signaling transduction mechanisms between the different *Hh* ligands, and that these defects are due to an insufficient response to secreted IHH in the cartilage. In addition, the disorganized retina in *rud* mutants also resembles that seen in embryos with an ablation of *Shh* using a *Thy1-Cre* [44]. The role of *Shh* and *Ihh* as mitogens in retinal neuroblast proliferation has been established [45], and *Shh*, *Ihh* and *Gli1* are expressed in retina at E12 and E13 [44], [46]. The difference in timing between the cortical and retinal defects (*rudolph* retinal molecular defects are seen at E14.5, but not E12.5, Figure S4) is consistent with the later expression pattern of *Shh* signaling components in the retina as compared to forebrain tissue.

The developing *rudolph* forebrain phenotype has both similarities to and differences from the known effects of *Shh* loss of function. The decreased cell proliferation and increased apoptosis we find are completely consistent with a role for *Shh* as a mitogen for the developing neural tissue and with the observations that blocking SHH function can lead to cell death [47], and that conditional ablation of *Smo* throughout the cortex by E9 using the *Foxg1-Cre* leads to increased cell death [48]. *Emx1-Cre* ablations of *Shh* and *Smo* in the dorsal telencephalon by E10 cause a smaller telencephalon featuring reduced proliferation and neuronal differentiation with increased cell death [49]. However, the striking disorganization of the cortex in *rudolph* mutants resembles more a loss of polarity phenotype, such as the cortical ablation of *numb* and *numb-like* [50]. Because *Shh* loss of function has not been demonstrated to directly affect polarity, we suggest that abnormalities of cortical signaling mechanisms other than *Shh* must be disrupted in the *rudolph* cortex to explain some of the developmental abnormalities of the CNS we observe. Because cortical dysplasia is not characteristic of any of the known human disorders of cholesterol biosynthesis, the extremely high CNS level of zymosterone, normally only a trace sterol in the brain, suggests that zymosterone or other 3-ketosterols in the *rud* brain could have a direct toxic effect or could otherwise impair neuronal differentiation.

### *Rudolph* is a hypomorphic allele of *Hsd17b7*

Two reports have recently described the phenotypes of *Hsd17b7* null allele mice [18], [19]. These embryos have major morphological abnormalities by E10.5, precluding direct comparison to the phenotypes studied here. The forebrain did appear smaller in the *Hsd17b7* homozygous null embryos, and development does not proceed past E9.5. A sterol analysis performed in these mutants demonstrated increased *Hsd17b7* enzyme substrates and unchanged cholesterol levels, similar to the results we report [19]. Maternal supply of cholesterol was also suggested to account for the normal embryonic cholesterol levels. Expression of *Shh* and *Ptc* was examined in the *Hsd17b7* homozygous null embryos at E8.5, but the domain and levels of expression did not differ from wild-type [19].

The phenotypic differences between the *rudolph* and *Shh* mutants may be due to the hypomorphic nature of the *rudolph* allele. However, we also note that the *rudolph* phenotype in the CNS begins to emerge around E12.5, which is the time when the blood-brain barrier forms and synthesis of cholesterol within the CNS becomes separated from non-neural cholesterol synthesis [51], [52], [53], [54]. The developmental consequences of reduced sterol concentrations in the early embryo could be mitigated by the maternal circulation in view of evidence that, in rodents, substantial amounts of maternal cholesterol can be transported to the fetus through the placental-fetal interface [55], thus possibly compensating for a lack of early *Hsd17b7* function in the initial patterning stages of embryonic development. We therefore propose that formation of the blood-brain barrier creates a neurodevelopmental compartment absolutely requiring endogenous *Hsd17b7* function, which, when absent, results in the severe phenotypes we describe here.

### Sterols in Hh signaling

Perhaps the most intriguing aspect of this study is that it is the first *in vivo* validation of several recent studies suggesting an intracellular role for sterols in SHH signaling [8], [11], [12]. These cholesterol intermediates have been demonstrated to have a role in the transduction of HH ligand signaling as well as the subcellular localization of HH signaling components. The fact that the mutant MEFs demonstrate normal Smoothened localization to the cilium, but a compromised response to SHH ligand, suggests that normal sterol concentrations are required for proper activation of Smoothened [6]. Alternatively, an inhibitory sterol may be present at increased levels, preventing the activation of the pathway.

Treatment with statins is a well-accepted method for lowering cholesterol levels in human patients by inhibiting *HMG-CoA reductase* (*Hmgcr*), the rate-limiting step in cholesterol biosynthesis. Statin treatment could also have significant effects on local concentrations of oxysterols generated from intermediates in the cholesterol biosynthetic pathway further downstream. Mouse *Hmgcr* mutants, which should genetically mimic a total block in the pathway at the site of action of statins, are not viable past implantation [56] and are therefore not informative in this regard. It will be important to study further the role of cholesterol intermediates and metabolites in various physiological settings and signaling paradigms. In doing so, we may find that statin treatments may be having unintended consequences in human health in sites of adult *Hh* activity, including adult neurogenesis.

## Materials and Methods

### Mouse husbandry and genotyping

*Rudolph* mice were originally generated by ENU mutagenesis of A/J mice and then outcrossed to FVB/J mice (both obtained from Jackson Labs, Bar Harbor, ME). Initial mapping was done with a whole genome SNP panel similar to one we previously described [17], and the mutation mapped to a 19.6 Mb interval on chromosome 1. Exon directed sequencing (including some flanking intronic sequence to identify mutations potentially affecting splicing) identified the *rudolph* mutation. Genotyping is done with either D1Mit454 or D1Mit524 microsatellite markers depending on strains involved. We maintained the colony with a combination of intercrossing and outcrossing to FVB. The C57BL/6J *Ptc1-lacZ* mouse was obtained from the Jackson laboratory and intercrossed with *rud* heterozygous mice;*Ptc1-lacZ* genotyping was done with standard lacZ primers. We have also performed backcrosses of the *rud* allele to mice on C57BL/6J and 129X1/SvJ backgrounds. All animals were housed in accordance with the Harvard Medical School ARCM regulations. Timed matings were checked for signs of copulation in the morning; vaginal plugs were noted and noon of that day was established as embryonic day (E) 0.5.

### Histology and immunohistochemistry

Embryos used for histological analysis were fixed with Bouin's fixative for at least forty-eight hours and processed for paraffin embedding using a Leica TP1020 automated tissue processor. Sections were cut at a thickness of 14 µm and stained with hematoxylin and eosin using standard techniques. Microscopy was done with a Leica DC500 or Zeiss AxioImager with ApoTome. TUNEL assay was performed with the In Situ Cell Detection Kit, TMR Red, following the manufacturer's instructions (Roche). BrdU labeling was done with a BrdU Labeling and Injection Kit (Roche). The TuJI antibody (SIGMA) was used at 1∶500 for 2 hours at room temperature on paraffin sections with citrate buffer antigen retrieval. Neural tube immunohistochemistry was performed using standard methods with antibodies from the Developmental Studies Hybridoma Bank.

### Skeletal measurements

To measure the size of the skeletal elements, embryos were stained for cartilage and bone using standard methods [57] and photographed. The length of each element was calculated using NIH Image J software, and units were converted to mm using standards.

### In vitro analysis of SHH signaling

Mouse embryonic fibroblasts were generated using standard methods and plated at a density of 20,000 cells/cm^2^ in the presence or absence of 200 ng/mL SHH amino terminal peptide (R&D Systems). Cell number was determined with the CyQuant Cell Proliferation Assay (Invitrogen), and β-galactosidase production was measured with the Galacto-Light Plus System (Applied Biosystems). Assays were performed 48 hours after plating. Cell growth experiments were done with an initial culture of 6,000 cells in a 96-well plate.

### RNAi clones

Lentiviral particles were made via transfection of 293T cells with plasmids including a plKO.1 control and a validated RNAi construct against mouse *Hsd17b7* (Open Biosystems, Huntsville, AL; clone TRCN0000041646). PzP53Med cells [20] were infected with 293T supernatant containing lentivirus. After puromycin selection, resistant cells were plated at clonal density and individual clones were isolated, maintained and analyzed with qRT-PCR for *Hsd17b7* levels. Control and knock-down clones were treated with SHH protein as described above.

### RT-PCR analysis

Total RNA from either brains or MEF cultures was prepared with TRIZOL (Invitrogen) and cDNA was made with qScript cDNA synthesis kit (Quanta) or the SuperScript RTIII system (Invitrogen). Hsd17b7 transcripts were analyzed with both random hexamer primed cDNA and gene specific primed cDNA synthesis (primer: TTTTGGTACCTCAGCTCGGGTGATCCGATTTCTG). *Hsd17b7* transcripts were analyzed with primers amplifying exons 6–8 (F: TCTGTATTCCAGTGTGATGTGC; R: CTTTTGGCCCGTGACGTAAT; 259 bp) or exons 6–7 (F: TCTGTATTCCAGTGTGATGTGC; R: CCACATTATGGGTAGGAGCAA ; 100 bp). Quantitative RT-PCR was done on a BioRad iCycler using either total RNA with Taqman probes (Applied BioSystems) or cDNA with Perfecta SYBR Green SuperMix (Quanta). SYBR-GREEN probes used were: *Ptc*-F (CCTGCAAACCATGTTCCAGTT ), *Ptc*-R (TCGTAGCCCCTGAAGTGTTCA) *Gli1*-F (CCAAGCCAACTTTATGTCAGGG), *Gli1*-R (AGCCCGCTTCTTTGTTAATTTGA), *Gapdh*-F (ACTCCACTCACGGCAAATTC), and *Gapdh*-R (TCTCCATGGTGGTGAAGACA).

### In situ hybridization and LacZ staining

Section mount in situ hybridization was done as previously described [58] with hybridization at 60 degrees and with BM Purple (Roche) for visualization of riboprobes. Probes used are published: *Gli1* [59] and *Ptc* [60]. Embryos were stained with lacZ using standard protocols [57] and then processed for paraffin histology as described above. Older embryos were fixed in 4% paraformaldehyde, cryoembedded in OCT, sectioned at 20 µm and stained on slides.

### Westerns

Embryos were homogenized in 1% SDS Lysis Buffer and protein extracts were run on a 10% polyacrylamide gel. A rabbit polyclonal antibody was used for Hsd17b7 (1∶1000, overnight at 4 degrees C), and a mouse monoclonal anti-actin antibody (1∶5000, SIGMA, 60 minutes at room temperature) was used as a loading control.

### GFP fusion proteins and transfections

Full-length mouse *Hsd17b7* from wild-type and mutant tissue was initially cloned into a pENTR/D/TOPO vector (Invitrogen) and then into the pcDNA-DEST47 vector (Invitrogen). DNA for either *Hsd17b7-GFP* or *rud-GFP* was co-transfected with Sec61β-mCherry (gift of T. Kirchhausen) into NIH3T3 cells using Fugene (Roche) following manufacturer's instructions. 48 hours after transfection, cells were fixed with 4% paraformaldehyde and counter-stained with DAPI. SMO localization within ciliated MEFs was observed by transfection of 20–30% confluent MEFs plated on 0.4% gelatin or poly-lysine coated coverslips in 24-well plates with the pBabePuro-A1∶Smo∶GFP plasmid ([9], gift of A. McMahon). Confluent cells were treated overnight with SHH peptide 48 hours after transfection. Immunocytochemistry was done by fixing with 4% paraformaldehyde/0.2 TritonX-100 for 20 minutes and blocking with 1% BSA for 60 minutes. Antibodies used were acetylated α-tubulin (SIGMA) at 1∶2000 for 60 minutes at room temperature and Goat anti-mouse Alexa Flour 594 (Invitrogen) at 1∶500 for 30 minutes at room temperature. Cells were mounted with VectaShield (Vector Laboratories) and imaged on a Zeiss AxioImager.

### Sterol analysis

Sterols were extracted from brain tissue as previously described with the addition of sterol-specific ions for the compounds of interest to this study [61]. Sterol levels are reported as a fraction of total sterols.

## Supporting Information

Figure S1 Variable Phenotypes of the *Rudolph* Mutant. In many mutants, the precursor to the nasal blood spot seen in late embryogenesis can be seen as a blebbing of the nasal epithelium as early as E12.5 (A). Introduction of the B6 genetic background to the colony resulted in some animals having more severe blebbing, which spreads beyond the tip of the snout (E18.5 in B, E12.5 in C). (E) The effect of the *rudolph* mutation on the splicing pattern of the *Hsd17b7* cDNA (PCR reaction spanning exons 6–8) does not change among tissues (heart/lung, limbs, or brain) from wild-type (w), heterozygous (*rud/+*; h) or mutant (*rud/rud*; m). cDNA obtained from embryos on the C57BL6 background (whole embryo: B6) or from B6 brain shows the same pattern of splicing.
(TIF)

Figure S2 Measurements of the long bones in both the forelimbs and hindlimbs at E16.5 and E18.5 show that mutant appendicular skeletal elements are significantly shorter at both stages.
(TIF)

Figure S3 *Rudolph* mutants have phenotypes throughout the CNS. Disorganized neural tissue is seen in the neural tube (B) at E16.5. Analysis of the forebrain at E12.5 shows grossly normal organization of the mutant brain (D) but some disorganization of cortical tissue is apparent (F).
(TIF)

Figure S4 The *rudolph* mutant form of Hsd17b7 protein is unstable. NIH3T3 cells were transfected with either wild-type *Hsd17b7-GFP* plasmid or a construct lacking the seventh exon to mimic the *rudolph* mutation (*rud-GFP*). Co-transfection with a Sec61-βmCherry plasmid serves to identify the endoplasmic reticulum (ER). Wild-type *Hsd17b7-GFP* is found in the (ER) as expected (A–C). Expression of the *rud-GFP* results in little or no GFP expression (D–F).
(TIF)

Figure S5 *Sonic hedgehog* signaling in *rudolph* mutants. *Patched-lacZ* expression in *rudolph*;*Ptc*-lacZ brain (B) is weaker at E14.5 than in wild-type;*Ptc*-lacZ embryos (A). *Gli1* expression in developing brain is also weaker in mutants (D) as compared to wild-type (C).
(TIF)

Figure S6 Neural tube patterning is normal in *rudolph* mutants. Immunohistochemical analysis in the neural tube at E10.5 (A–N) and E12.5 (O–BB) for a variety of cell fates along the dorsal-ventral axis show no changes in patterning between wild-type and *rudolph* mutant embryos.
(TIF)

Table S1 Long bone and digit length. Skeletal preparations of wild-type and *rudolph* embryos were prepared and the length of the skeletal elements were measured in Image J and converted to mm.
(DOC)

Table S2 Mutant and phenotype incidence. Embryos were harvested from the genetic backgrounds indicated and associated phenotypes quantified.
(DOC)
