# Supplementary figures and images for: Cholesterol Metabolism Is Required for Intracellular Hedgehog Signal Transduction In Vivo
Source: PLoS Genet. 2011 Sep 1;7(9):e1002224. doi: 10.1371/journal.pgen.1002224 (PMC3164675; doi:10.1371/journal.pgen.1002224)

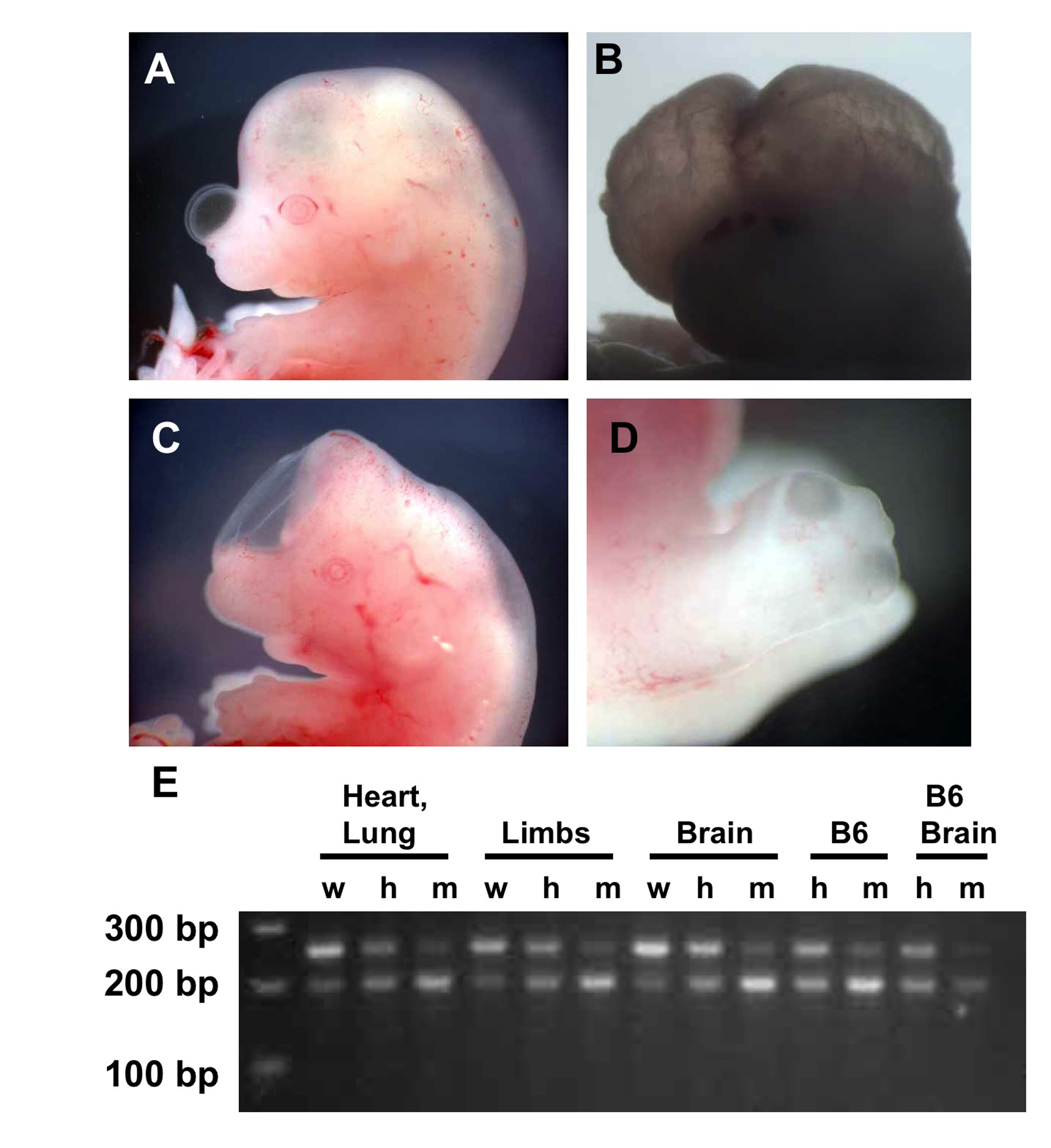

Supplement: Figure S1 — Variable Phenotypes of the Rudolph Mutant. In many mutants, the precursor to the nasal blood spot seen in late embryogenesis can be seen as a blebbing of the nasal epithelium as early as E12.5 (A). Introduction of the B6 genetic background to the colony resulted in some animals having more severe blebbing, which spreads beyond the tip of the snout (E18.5 in B, E12.5 in C). (E) The effect of the rudolph mutation on the splicing pattern of the Hsd17b7 cDNA (PCR reaction spanning exons 6–8) does not change among tissues (heart/lung, limbs, or brain) from wild-type (w), heterozygous (rud/+; h) or mutant (rud/rud; m). cDNA obtained from embryos on the C57BL6 background (whole embryo: B6) or from B6 brain shows the same pattern of splicing. (TIF) [file pgen.1002224.s001.tif]

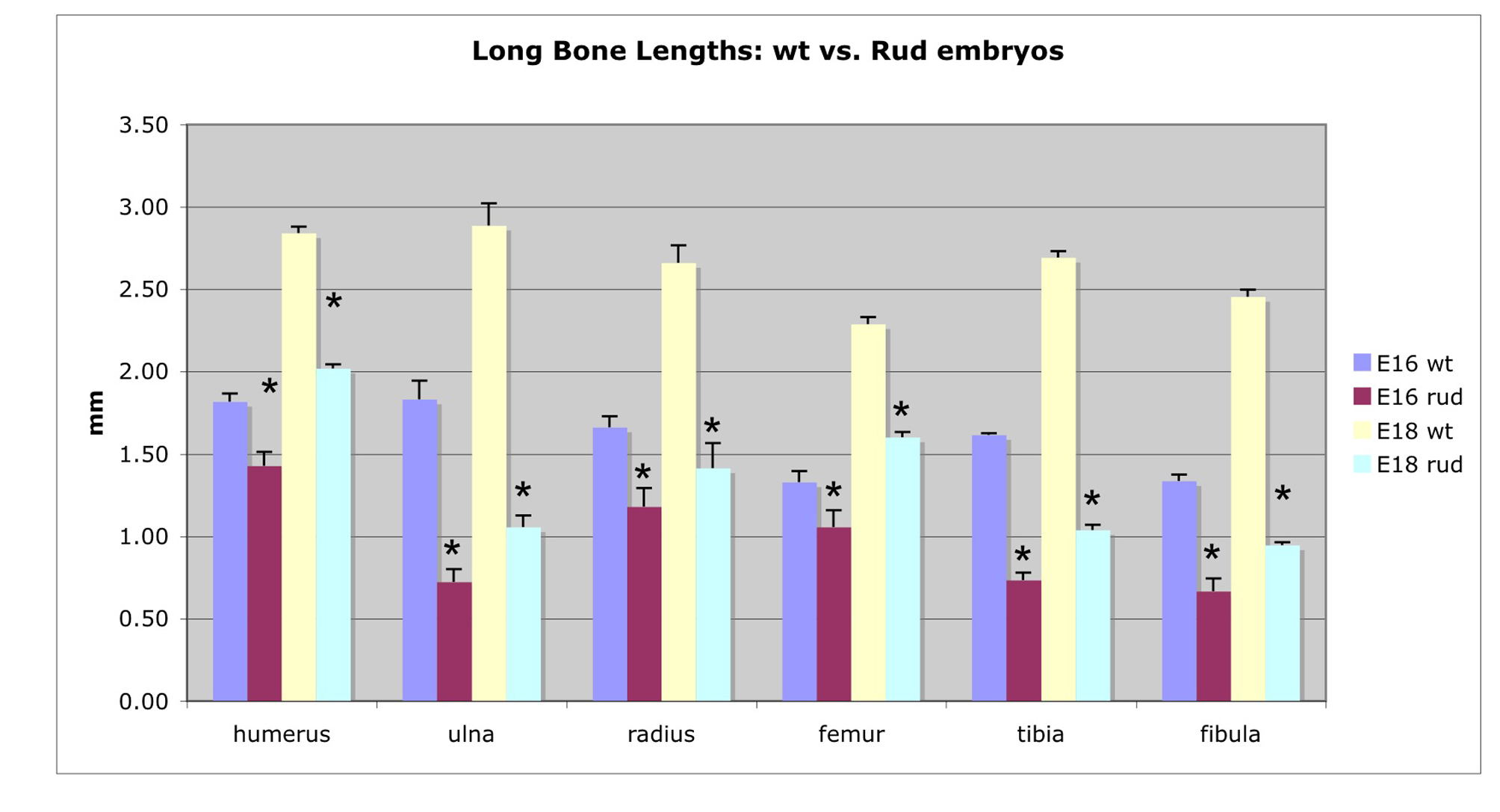

Supplement: Figure S2 — Measurements of the long bones in both the forelimbs and hindlimbs at E16.5 and E18.5 show that mutant appendicular skeletal elements are significantly shorter at both stages. (TIF) [file pgen.1002224.s002.tif]

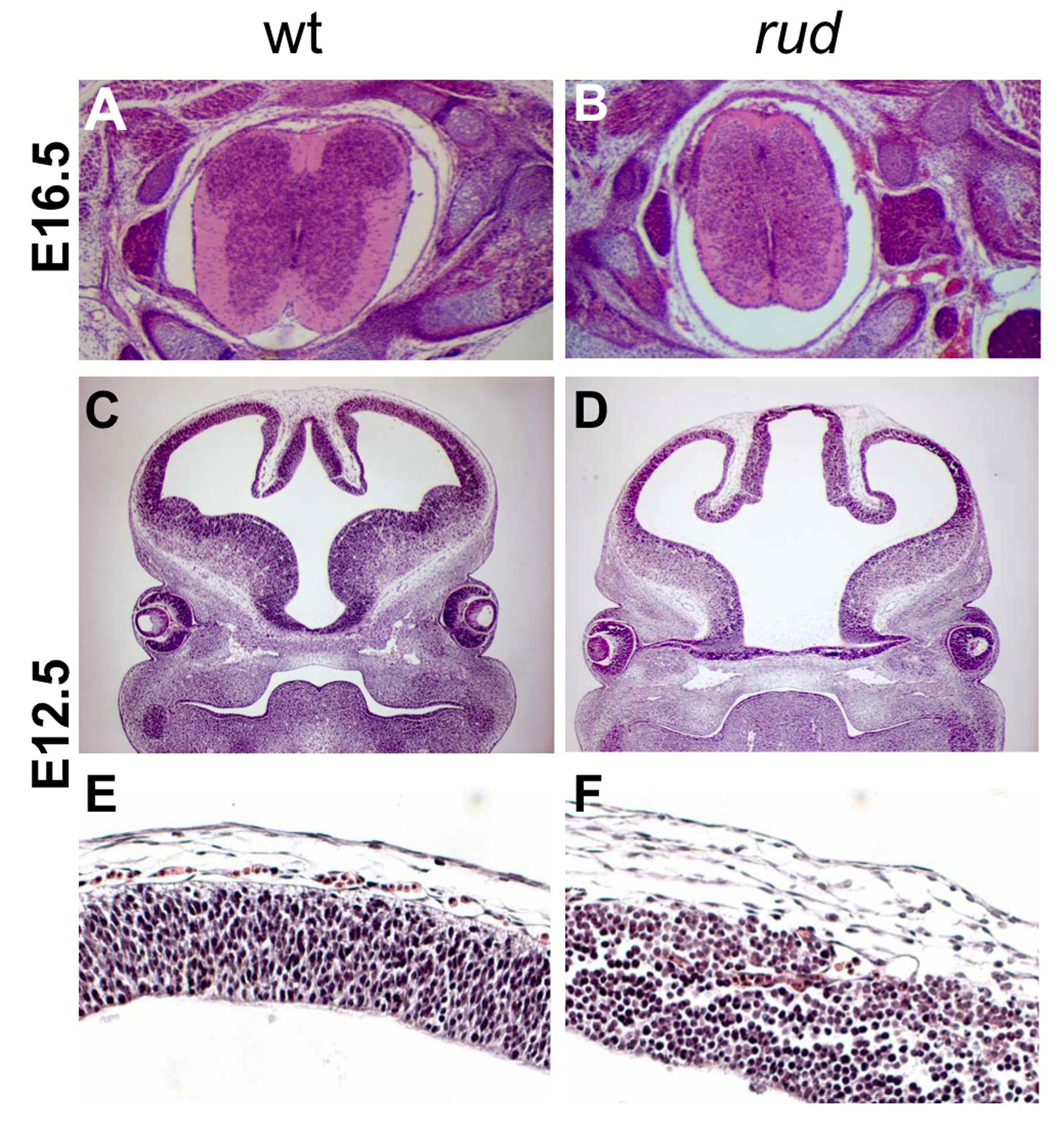

Supplement: Figure S3 — Rudolph mutants have phenotypes throughout the CNS. Disorganized neural tissue is seen in the neural tube (B) at E16.5. Analysis of the forebrain at E12.5 shows grossly normal organization of the mutant brain (D) but some disorganization of cortical tissue is apparent (F). (TIF) [file pgen.1002224.s003.tif]

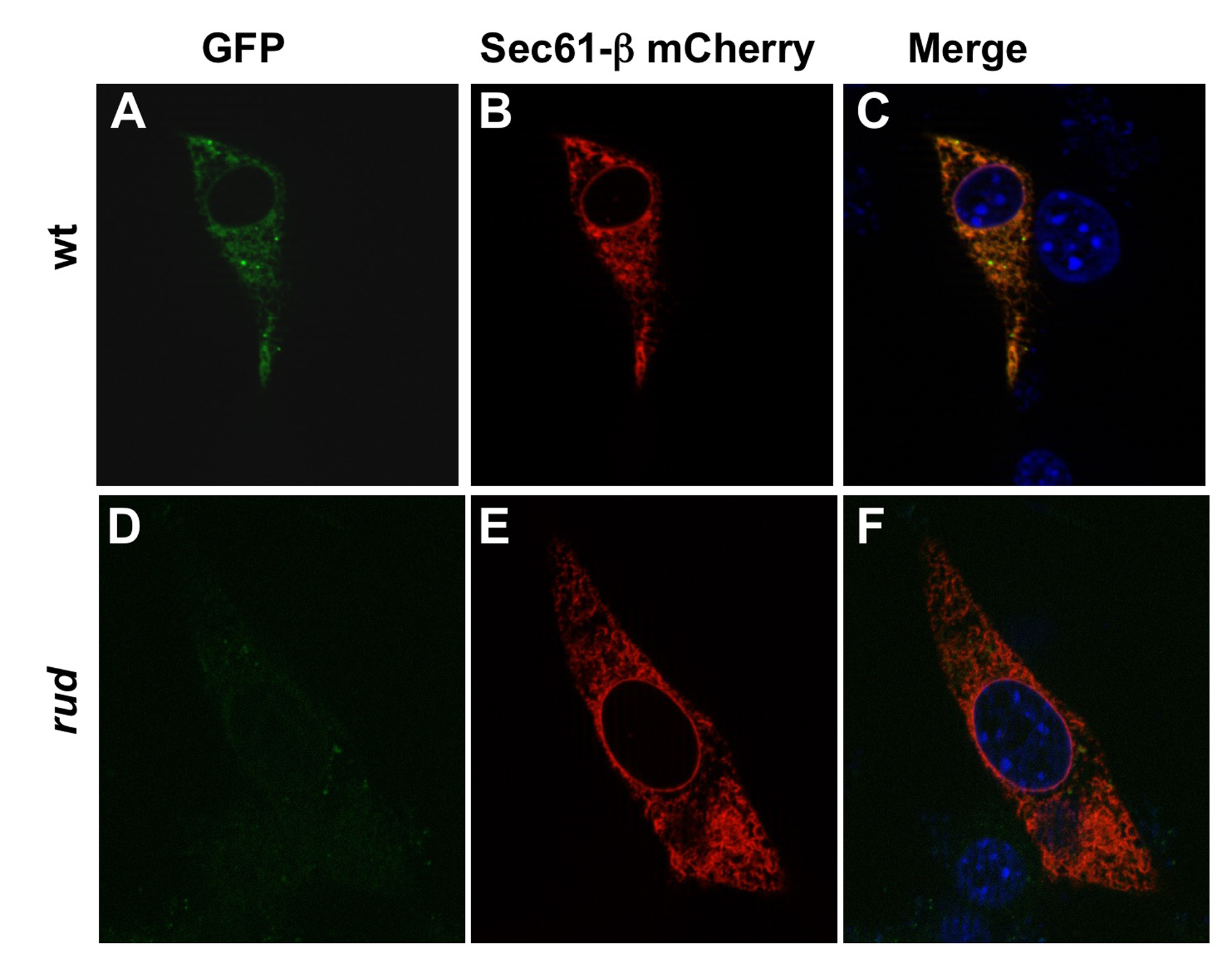

Supplement: Figure S4 — The rudolph mutant form of Hsd17b7 protein is unstable. NIH3T3 cells were transfected with either wild-type Hsd17b7-GFP plasmid or a construct lacking the seventh exon to mimic the rudolph mutation (rud-GFP). Co-transfection with a Sec61-βmCherry plasmid serves to identify the endoplasmic reticulum (ER). Wild-type Hsd17b7-GFP is found in the (ER) as expected (A–C). Expression of the rud-GFP results in little or no GFP expression (D–F). (TIF) [file pgen.1002224.s004.tif]

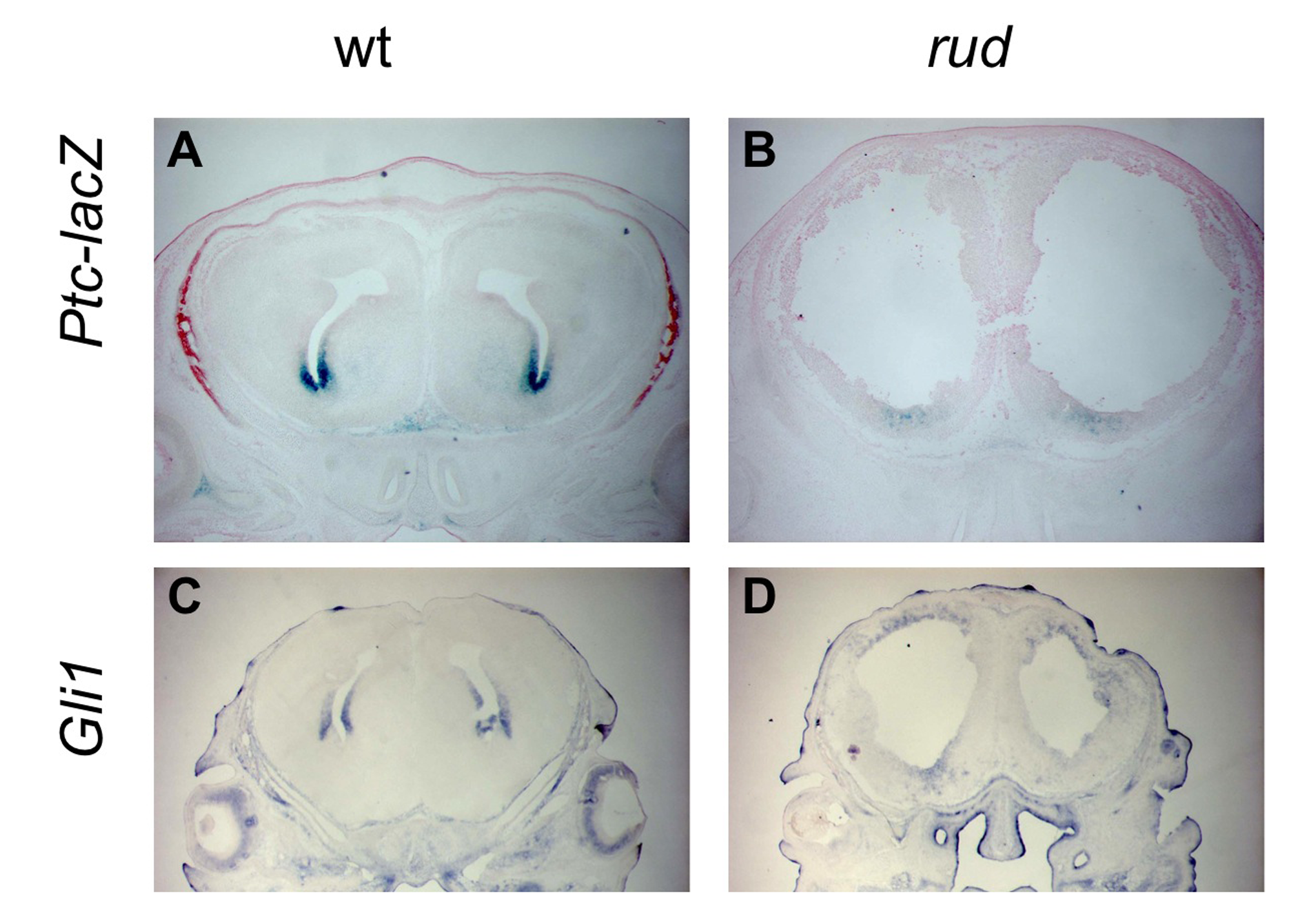

Supplement: Figure S5 — Sonic hedgehog signaling in rudolph mutants. Patched-lacZ expression in rudolph;Ptc-lacZ brain (B) is weaker at E14.5 than in wild-type;Ptc-lacZ embryos (A). Gli1 expression in developing brain is also weaker in mutants (D) as compared to wild-type (C). (TIF) [file pgen.1002224.s005.tif]

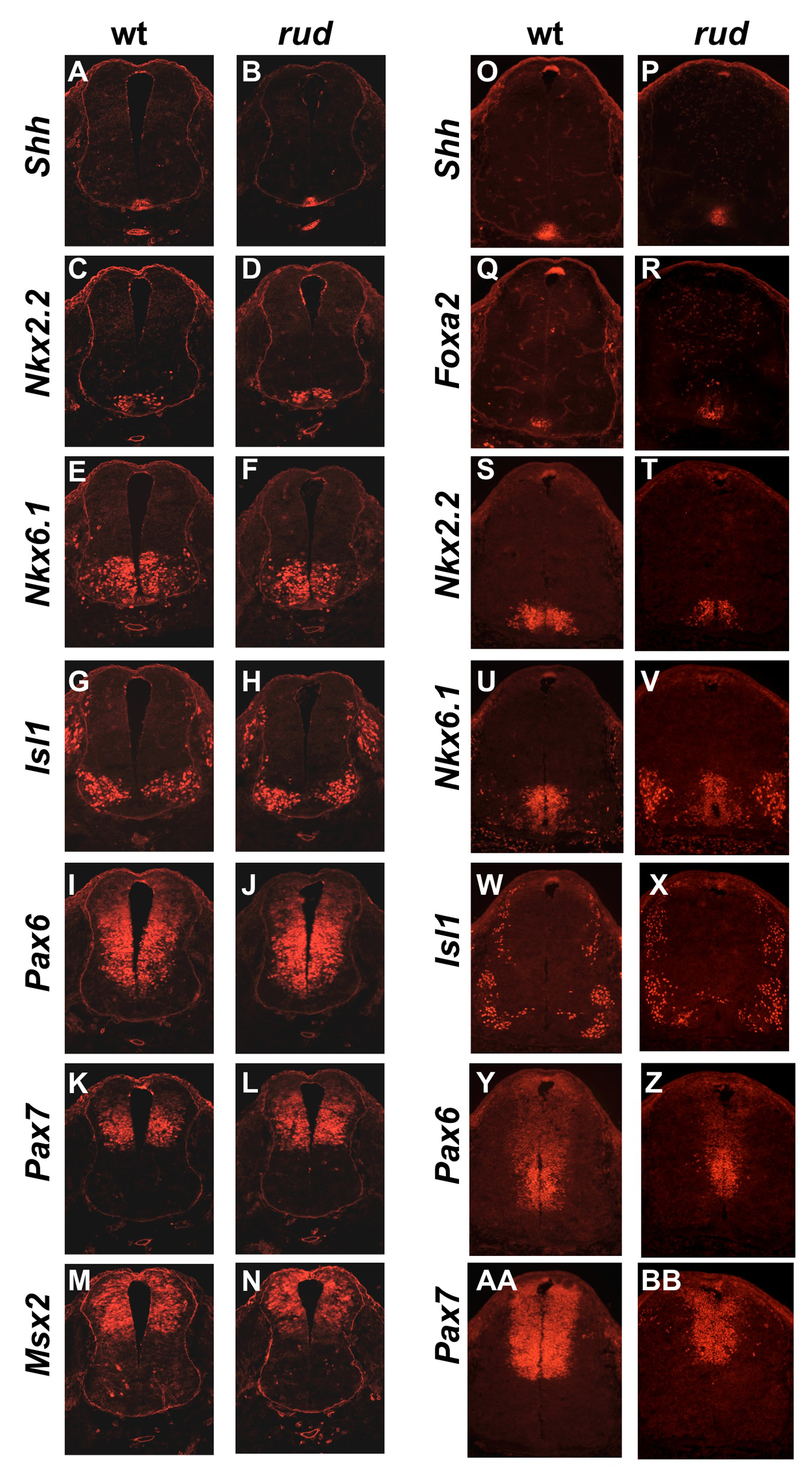

Supplement: Figure S6 — Neural tube patterning is normal in rudolph mutants. Immunohistochemical analysis in the neural tube at E10.5 (A–N) and E12.5 (O–BB) for a variety of cell fates along the dorsal-ventral axis show no changes in patterning between wild-type and rudolph mutant embryos. (TIF) [file pgen.1002224.s006.tif]
