# Supplementary material for: Cholesterol Metabolism Is Required for Intracellular Hedgehog Signal Transduction In Vivo
Source: PLoS Genet. 2011 Sep 1;7(9):e1002224. doi: 10.1371/journal.pgen.1002224 (PMC3164675; doi:10.1371/journal.pgen.1002224)
Supplement: Table S1 — Long bone and digit length. Skeletal preparations of wild-type and rudolph embryos were prepared and the length of the skeletal elements were measured in Image J and converted to mm. (DOC) [file pgen.1002224.s007.doc]

# Table SI. Long bone and digit length

**E16.5 E18.5**

**wt *rud* wt *rud***

**humerus** 1.82+0.05 1.43+0.09 2.84+0.04 2.02+0.02

**radius** 1.66+0.07 1.18+0.11 2.66+0.10 1.41+0.15

**ulna** 1.83+0.11 0.72+0.08 2.89+0.14 1.05+0.07

**femur** 1.33+0.07 1.06+0.10 2.29+0.04 1.60+0.03

**tibia** 1.62+0.01 0.73+0.05 2.69+0.04 1.04+0.03

**fibula** 1.33+0.04 0.67+0.08 2.46+0.04 0.95+0.02

**Forelimb**

**Digit** **I** 1.113+0.61 0.962+0.51 1.464+0.45 1.414+0.58

**Digit II** 1.864+0.57 1.807+0.93 2.190+0.33 2.191+0.44

**Digit III** 2.003+0.53 1.890+0.63 2.213+0.38 2.289+0.51

**Digit IV** 1.951+0.51 1.911+0.72 2.293+0.48 2.301+0.68

**Digit V** 1.313+1.16 1.433+0.67 1.614+0.88 1.878+0.88

**Hindlimb**

**Digit I** 0.644+0.36 0.603+0.22 0.655+0.22 0.720+0.26

**Digit II** 1.278+1.02 1.335+0.40 1.649+0.64 1.618+0.31

**Digit III** 1.464+0.98 1.509+0.42 1.868+0.99 1.765+0.44

**Digit IV** 1.363+0.72 1.451+0.41 1.736+0.78 1.646+0.64

**Digit V** 0.958+1.17 1.083+0.41 1.318+0.70 1.117+0.53

Skeletal preparations of wild-type and *rudolph* embryos were prepared

and the length of the skeletal elements were measured in Image J and converted to mm.
