# Supplementary material for: Cholesterol Metabolism Is Required for Intracellular Hedgehog Signal Transduction In Vivo
Source: PLoS Genet. 2011 Sep 1;7(9):e1002224. doi: 10.1371/journal.pgen.1002224 (PMC3164675; doi:10.1371/journal.pgen.1002224)
Supplement: Table S2 — Mutant and phenotype incidence. Embryos were harvested from the genetic backgrounds indicated and associated phenotypes quantified. (DOC) [file pgen.1002224.s008.doc]

**Table S2. Mutant and Phenotype Incidence**

**Genetic Mutants Recovered Blebs Limb Dead**

**Background Expected Observed beyond nose Defects Mutants**

AJ/ FVB 18 15 0 0 0

AJ/ FVB/ B6 131 115 17(14.8%) 12(10.4%) 9 (7.8%)

N1,N2,N3 B6 35 33 16(48.5%) 1 (3.0%) 4(12.1%)

N3 FVB 13 14 0 0 0

N1,N2 129 4 2 0 0 1

Embryos were harvested from the genetic backgrounds indicated and associated phenotypes quantified.
